# The 3D architecture of a bacterial swarm has implications for antibiotic tolerance

**DOI:** 10.1038/s41598-018-34192-2

**Published:** 2018-10-25

**Authors:** Jonathan D. Partridge, Gil Ariel, Orly Schvartz, Rasika M. Harshey, Avraham Be’er

**Affiliations:** 1 Department of Molecular Biosciences, University of Texas at Austin, Austin, Texas 78712 USA; 2 Department of Mathematics, Bar-Ilan University, Ramat Gan, 52000 Israel; 3 Zuckerberg Institute for Water Research, The Jacob Blaustein Institutes for Desert Research, Ben-Gurion University of the Negev, Sede Boqer Campus, 84990 Midreshet Ben-Gurion, Israel; 4 Department of Physics, Ben-Gurion University of the Negev, 84105 Beer Sheva, Israel

## Abstract

Swarming bacteria are an example of a complex, active biological system, where high cell density and super-diffusive cell mobility confer survival advantages to the group as a whole. Previous studies on the dynamics of the swarm have been limited to easily observable regions at the advancing edge of the swarm where cells are restricted to a plane. In this study, using defocused epifluorescence video imaging, we have tracked the motion of fluorescently labeled individuals within the interior of a densely packed three-dimensional (3D) region of a swarm. Our analysis reveals a novel 3D architecture, where bacteria are constrained by inter-particle interactions, sandwiched between two distinct boundary conditions. We find that secreted biosurfactants keep bacteria away from the swarm-air upper boundary, and added antibiotics at the lower swarm-surface boundary lead to their migration away from this boundary. Formation of the antibiotic-avoidance zone is dependent on a functional chemotaxis signaling system, in the absence of which the swarm loses its high tolerance to the antibiotics.

## Introduction

The motion of individual flagellated bacteria swimming in bulk liquid is fairly well understood. In some species, the normal run-tumble random walk motion is controlled by a chemosensory signal transduction pathway, which biases this walk to migrate towards favorable environments such as a nutrient source, or away from toxic environments^1–3^. However, bacterial motion is not limited to swimming in bulk liquid and can also occur on a surface. An example of surface motion is ‘swarming’, where large numbers of flagellated bacteria migrate as a dense consortium. A swarming colony is distinct from a biofilm, which by definition consists of a mass of metabolically quiescent cells attached firmly to a surface. In contrast, bacteria in a swarm are motile, unattached, and metabolically active^4–6^. Efficient expansion of a swarm is promoted in many swarming species by secreted surfactants that decrease surface tension. The bacteria move in packs within a thin layer of liquid present on the surface, and display an intricate swirling motion where hundreds of dynamic bacterial clusters continuously form and dissociate as the bacterial mass moves forward, colonizing evermore territory. Quantitatively, the intricate patterns depend on the species under study, culture conditions, external stimuli, surface hydration, and/or run-tumble bias, and stem from both strong, short-range, steric repulsion as well as long-range hydrodynamic interactions^7–14^. The flagellar mechanics during swarming are expected to be similar to swimming, i.e. flagellar motors rotate bi-directionally to produce runs and tumbles (or reversals), except that the crowded environment and/or the altered cell physiology of the swarm may enforce a largely forward motion^7,10^. Swarming not only helps bacteria to colonize new niches, but has also been demonstrated to facilitate their survival on antibiotic concentrations that are lethal to the same bacteria while swimming in liquid^15–19^. Dense suspensions of swimming bacteria, obtained by artificially concentrating the cells to high volume fractions, exhibit collective motion as well^20–26^, but these bacteria are not equivalent to the swarm collective because swarming bacteria have an altered physiology^5,6^. How bacteria move within the swarm, if they respond to chemical gradients, and whether their motion contributes to their survival, is poorly understood compared to their swimming counterparts.

A bacterial swarm typically consists of a monolayer of cells at an advancing edge, with gradually increasing bacterial density behind, where multiple layers of cells are evident. The motion of bacteria near the edge in quasi two-dimensional space (1–2 cells deeps; referred to here as 2D) has been extensively analyzed^7–14^. The motion of cells swimming in bulk liquid in 3D (referred to as 3D swimming) has also been well studied^27–29^. Unlike swarming, swimming cells populate the bulk liquid sparsely, and move without constraints imposed by neighboring bacteria^5,6^. No attempt has yet been made to track the motion of bacteria in the 3D space of a densely packed swarm. The architecture of this region is expected to be complex due to the strong interactions arising from confinement of the cells in the crowd, their interaction with the surrounding viscoelastic, non-Newtonian liquid, as well as cell-surface interactions at two different boundaries – the solid-liquid interface at the bottom, and the liquid-air interface at the top of the swarm. It is known that the dense mass of cells in a swarm is critical to its capacity for antibiotic tolerance^18^. Two key questions we hope to address are: (i) how do bacteria optimize their use of the 3D space? and (ii) does the bacterial distribution shed light on the known ability of swarms to survive otherwise lethal antibiotic concentrations? Analysis of a 3D swarm is expected to reveal new principles of collective motion with important ecological and medical implications.

In this study, we have used defocused epifluorescence video imaging to track the motion of fluorescently labeled individuals within a 3D region of a *Serratia marcescens* swarm. We find an unusual distribution of cells, with non-trivial dynamics close to the bottom and top boundaries. At the top, bacteria stay away from the air boundary, a pattern correlated with the presence of a secreted biosurfactant at this boundary. At the bottom, the bacteria avoid the solid agar boundary only when antibiotics are introduced into the medium. Surprisingly, the avoidance response is dependent on a functional chemosensory pathway, a response not reported to occur in bacteria swimming in 3D. In the absence of this pathway, the cells no longer avoid the bottom boundary, and lose the resistance to antibiotics conferred by the swarm. Our analysis indicates that the dynamics of swarming bacteria are guided by both physics and biology, each informing different facets of the bacteria throughout the 3D structure.

## Results

### Strategy for monitoring cell movement in a 3D region of the swarm

**Figure 1:**
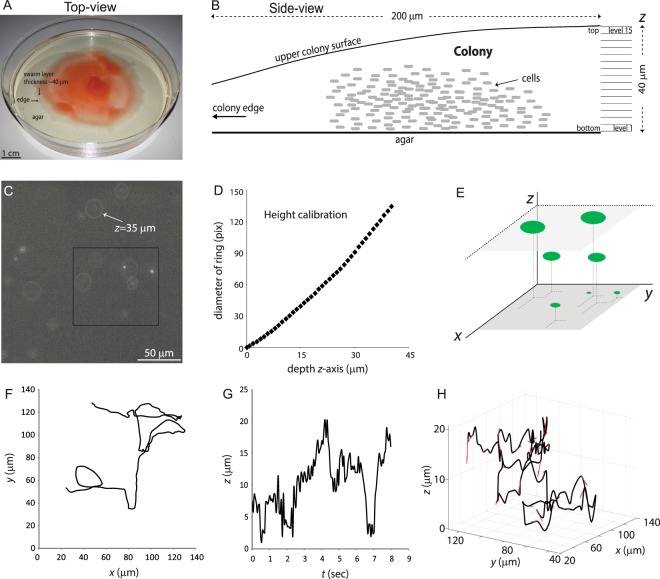
Tracking 3D trajectories of fluorescent bacteria in a multilayered swarm using defocused imaging (DI) methodology. (**A**) A macroscopic view of a *S. marcescens* swarming colony inoculated in the center of the agar plate and allowed to swarm out. The region observed for tracking was ~100–400 μm behind the advancing edge (downward pointing arrow). (**B**) A sketch of the cross section of the colony near the edge, illustrating the approximate shape and dimensions in this region. The height of the colony at the point of observation was estimated to be ~40 μm (see Methods). This height was arbitrarily divided into 15 levels or bins. (**C**) A snapshot of off-focus fluorescent images viewed from the top. The diameters of the diffraction rings report on bacterial location at different heights in the colony. Arrow points to an example of a relatively large diffraction ring corresponding to location of a cell 35 μm above the agar. There are, on average, 1000 times more unlabeled non-fluorescent cells in any view of the fluorescence image as determined by viewing the same field in phase contrast (see Movie S4). (**D**) The calibration curve correlating the diameter of the diffraction ring as a function of the depth of the cells, as described in Methods. (**E**) A cartoon showing how height is estimated from the size of the diffraction ring for bacteria in C. (**F**) The *x-y* trajectory of a typical cell moving inside the 3D colony. (**G**) The height of the same cell plotted in F as a function of time. (**H**) The 3D trajectory of the same cell plotted in (**F,G**). Small red arrows indicate instantaneous vectors of the torsion and curvature of the trajectory (see Methods).

We chose *S. marcescens* for our studies because this bacterium swarms efficiently and reliably, likely because it secretes copious amounts of the surfactant serrawettin^11,30^. We have used this bacterium before for 2D swarm analysis at the edge region of the swarm, where the colony height is ~3–4 μm and cells are 1–2 layers deep (see^10^ and Movie S1). As done in the previous 2D analysis^10^, bacteria expressing a green fluorescence protein (GFP) were mixed with non-fluorescent cells at a ratio of ~1:1000 and inoculated together on the swarm plate, in order to track the motion of the fluorescently labeled individuals within the dense pack of unlabeled cells. In the present study however, we tracked the bacteria in the multilayered 3D region of the swarm, ~100–400 μm behind the advancing edge (Fig. 1A). The colony height in this region was ~40 μm, fairly constant with respect to the distance from the edge, and highly reproducible in repeat experiments (Fig. 1A,B; sample-to-sample differences in the colony height was smaller than 5 μm). The swarm was viewed from the top, and the position of the fluorescent cells was determined using a defocused imaging (DI) technique first used to track fluorescent nanoparticles^31^. This method was recently used to track bacteria swimming in 3D, where cell density is sparse and cells can be viewed directly using phase contrast^27,28^. The DI method uses the diameter of the largest observed diffraction ring as a linear measure of an object’s distance |*z*| from the focal plane. Movie S2 shows a typical example of the motion of the fluorescent cells using this method, and a snapshot of the screen is shown in Fig. 1C. The observed ring diameters were translated into the *z* positions of the cells (Fig. 1D), as described under Methods. A segment of the instantaneous position of some of the cells seen in Fig. 1C is sketched in Fig. 1E. Different projections of an example cell trajectory are plotted in Fig. 1F–H (Movie S3).

For simplicity, we divided the 40 μm colony height into 15 bins (levels), each approximately 2.66 μm in height (equal to ~3 layers of cells stacked on their short side) (Fig. 1B). Level 1 is at the bottom near the agar boundary, and level 15 is at the top near the air boundary. In the first set of tracking experiments, which we refer to as ‘normal conditions’, we followed fluorescent wild-type (WT) cells on a standard swarm plate without any additions or modifications. In other sets, various mutant strains and conditions were tested: these included chemotaxis mutants (labeled Che^-^) that are unable to respond to attractant or repellent chemical gradients, a mutant defective in production of serrawettin (labeled Srw^-^), and additions of antibiotics [Kanamycin (inhibits protein synthesis), and Ciprofloxacin (inhibits DNA gyrase and TopoIV, and ultimately cell division)], referred to as Kan and Cipro). In each case, fluorescently labeled strains were tracked in a mixture with their unlabeled counterparts (Table S1 lists all strains used).

### Structure of a wild-type swarm

**Figure 2:**
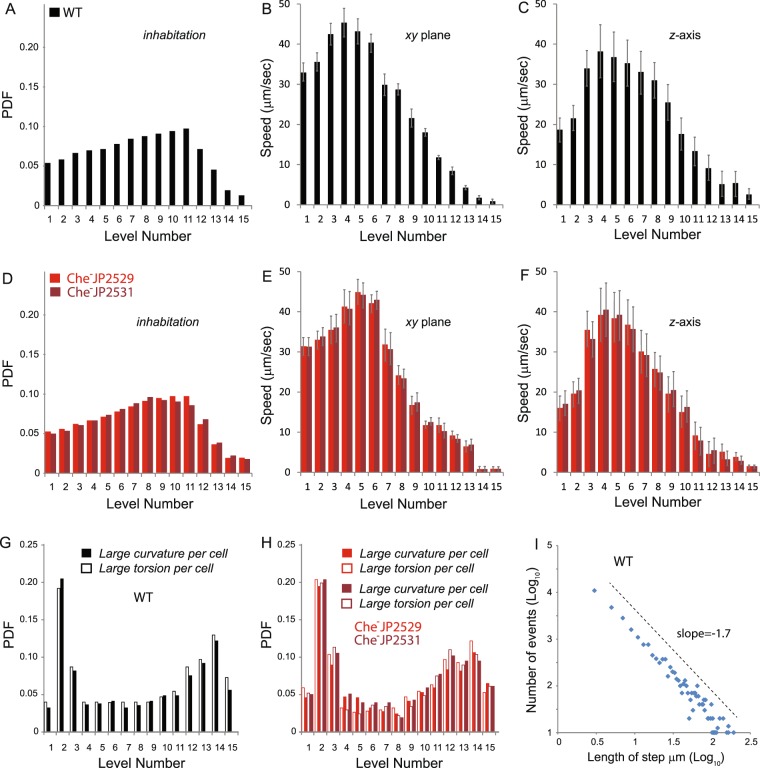
Inhabitation, speeds and turning rates of WT and Che- bacteria in a 3D swarm. The swarms contain a mixture of fluorescently-labeled and unlabeled cells. The fluorescent cells were tracked by DI as described in Fig. 1C–H. **(A,D)** The occupancy of WT (JP1020) and Che^-^ mutants (JP2529 and JP2531) across 15 levels in a region whose maximal colony height is ~40 μm (see Fig. 1B). PDF stands for probability density function, where the *Y*-axis is normalized to a sum of all the data points (>200,000). **(B,E)** The average speed in the *x-y* plane at different heights for cells tracked in A and D, respectively. **(C,F)** The average speed along the *z*-direction at different heights for cells tracked in A and D, respectively. Speeds were calculated using a Matlab program as described under Methods. Error bars indicate standard deviations from the mean. **(G,H)** PDF of curvatures and torsions for WT **(**>250,000 data points) and Che^-^ mutants (>200,000 data points). See Movie S5. **(I)** The graph plotting the steps between significant curvatures and torsions events for WT yields a power law distribution. See the section on ‘Curvature and torsion of trajectories’ under Methods for a fuller discussion of the results in G-I.

Trajectories of >2000 WT bacteria (obtained as described in Fig. 1D–H) growing under ‘normal conditions’ (as described above) were used to obtain the instantaneous position of the cells, yielding the average fraction of time cells spend in each level (Fig. 2A). Each bacterium yielded hundreds of data points. The cell population exhibited a smooth and moderate positive increment with increasing height from the bottom agar boundary, dropping sharply close to the top. Thus, the maximum colony density is not at the top but below it, at approximately two-thirds the distance from the bottom. Near the top, cells were sparse, with the top two levels containing very few cells. Next, we plotted the instantaneous speed of the cells in both the *x-y* plane and along the *z*-direction as described under Methods (Fig. 2B,C). The maximal cell speed was recorded at intermediate heights, with slightly lower speeds near the bottom, and significantly lower speeds near the top. The most intensive collective motion of the swarm occurred in levels 3–5, far below the top (Movie S4). Some cells became seemingly immobilized at the top (levels 13–15; see Movie S2), indicating perhaps the presence of material other than fluid in the upper level. However, after a few seconds, such cells would migrate down back to the colony, where they moved normally like others in this region. Finally, we also quantified the turning rate of cells in terms of the curvature and torsion of trajectories (Fig. 2G,I; Movie S5; see Methods). The results indicate that the cells change their direction when close to the boundaries and tend to move in relatively straight lines in the bulk.

### The structure of a swarm is not dictated by chemotaxis

The non-uniform distribution of cells within a 3D swarm (Fig. 2A) could be a reflection of chemotaxis, with cells migrating to an optimal nutrient or oxygen zone, assuming such chemical gradients exist. To verify the role of chemotaxis in the 3D inhabitation profile, we used two versions of a non-chemotactic (Che^−^) mutant that is unable to respond to chemical gradients. WT bacteria perform chemotaxis by modulating their run-tumble motion, which is regulated by many genes in the chemotaxis signaling pathway, and dictated by the reversal frequency of the bi-directionally rotating flagellar motor (Fig. S1A)^1,2^. JP2529 is *S. marcescens* missing a gene (*cheY*) whose product normally promotes flagellar motor reversals, resulting in bacteria with no motor reversals but normal motor speeds (Fig. S1B). These bacteria can only run and cannot perform chemotaxis (Fig. S1C). JP2531 is a version of this strain manipulated to produce motor reversals so that it has a basal run-tumble bias, by introduction of a plasmid (pXYZ202) that provides a constitutively active form of CheY (CheY**; Fig. S1B); this mutant remains non-chemotactic (Fig. S1C). Both mutant strains swarmed well under our experimental conditions (see Methods). Analysis of the 3D structures of both Che^−^ strains showed essentially a WT pattern of habitation, as well as a WT distribution of cell speeds and trajectories (Fig. 2D–F,H). Thus, the distribution pattern of bacteria in a normal 3D *Serratia* swarm is not mediated by chemotaxis or the requirement for a run-tumble bias.

### Presence of surfactant is correlated with the depletion of cells near the top of the swarm

**Figure 3:**
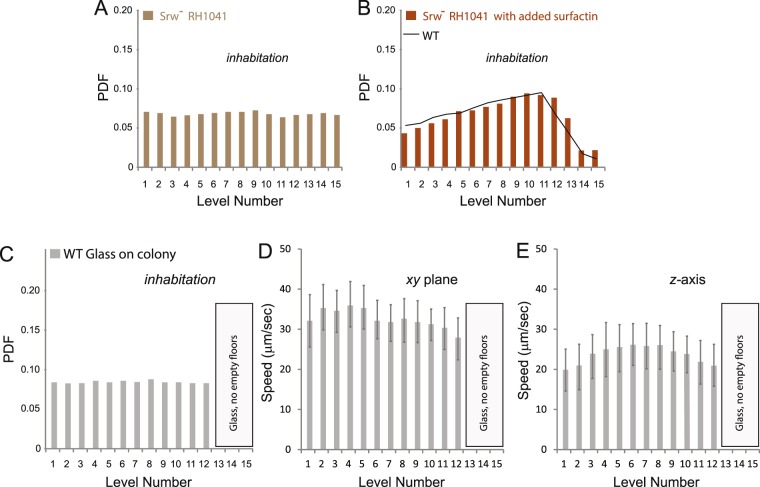
Inhabitation of Srw- bacteria in a 3D swarm. The swarms contain a mixture of fluorescently-labeled and unlabeled cells. The fluorescent cells were tracked by DI as described in Fig. 1C–H. (**A**) A Srw^-^ mutant of *S. marcescens* (RH1041) is distributed uniformly across the height of the colony. In (**B**), a commercial source of surfactin from *B. subtilis* was added to the Srw^-^ mutant, recovering the WT cell occupancy pattern (black trace). (**C**) A glass cover slip (1 × 1 cm, 100 μm in thickness) was placed gently on the top of the WT colony without disturbing the swirling motion underneath. Cell distribution changed to that seen in the Srw^-^ strain in A. PDF values represent >100,000 data points in each figure. (**D,E**) The average speed in the *x-y* and *z* planes at different heights for cells tracked in C, respectively, calculated as described in Fig. 2. PDF, probability density function (see Fig. 2 legend).

*S. marcescens* secretes large amounts of serrawettin, an amphiphilic molecule that is thought to aid swarming by reducing surface friction and surface tension. Such molecules are produced by a number of swarming species, including *Bacillus subtilis*, which makes a surfactant called surfactin^6^. Given that a large zone of serrawettin and surfactin is present ahead of the swarm edge of *S. marcescens* and *B. subtilis*, respectively^11^, these surfactants are expected to coat the colony as well, i.e. be present on top of the colony. To test if the lack of cell habitation at the top of a WT swarm was due to the presence of serrawettin, we evaluated the behavior of a Srw^−^ mutant (RH1041; Fig. 3A). This mutant swarmed well under our experimental conditions (see Methods). There was a striking change in the 3D structure of the Srw^−^ swarm, where the bacteria were now distributed uniformly at all levels i.e. the cell-depletion zone occupying ~levels 13–15 present in WT, disappeared in the Srw^−^mutant (compare Figs 3A and 2A). To test if this new cell distribution pattern was related to loss of surfactant, we added back surfactin produced by *B. subtilis* (see Methods), which is similar in structure to serrawettin^5^. Surfactin addition shifted the distribution of cells back to the WT mode, i.e. loss of habitation in the upper levels (Fig. 3B). To confirm this result independently, we also looked at the 3D structure of swarms of *Escherichia coli* and *Salmonella enterica* – bacteria that do not produce surfactant^32^ (Fig. S2); the structure of their swarms was similar to the Srw^−^ *S. marcescens* shown in Fig. 3A. We conclude that the cell depletion zone in a WT swarm is due to the presence of serrawettin.

### Glass can displace the serrawettin layer on top of the swarm

To further probe whether absence of bacteria at the top of the colony is indeed related to presence of the surfactant, we gently placed a glass cover slip on the surface of a WT swarm in a manner that did not disrupt swarming, i.e. cells continued to exhibit intense swirling motion when observed under the microscope. This was not surprising, as even *E. coli* transferred from a swarm and placed between glass and a PDMS membrane have been shown to continue swarming^33^. We observed that placement of the glass changed the 3D structure of the WT swarm such that it now resembled that of a Srw^−^ swarm, in that cells exhibited a uniform distribution, inhabiting all levels (Fig. 3C). This suggests that the glass displaced the surfactant layer. Interestingly, the swarmers did not congregate near the glass, and showed a speed distribution that was independent of the distance from the glass (Fig. 3D–E). This observation was unexpected because similar experiments with bacteria swimming in a drop of liquid have reported congregation of swimming cells near glass, with bacterial speeds varying depending on their distance from the glass [e.g.^29^]. This behavior of swimmer cells has been interpreted to be the result of hydrodynamic interactions of bacteria with glass. Clearly, the swarmer cells have a different response to the same material.

### Bacteria avoid the bottom agar boundary when antibiotics are present

**Figure 4:**
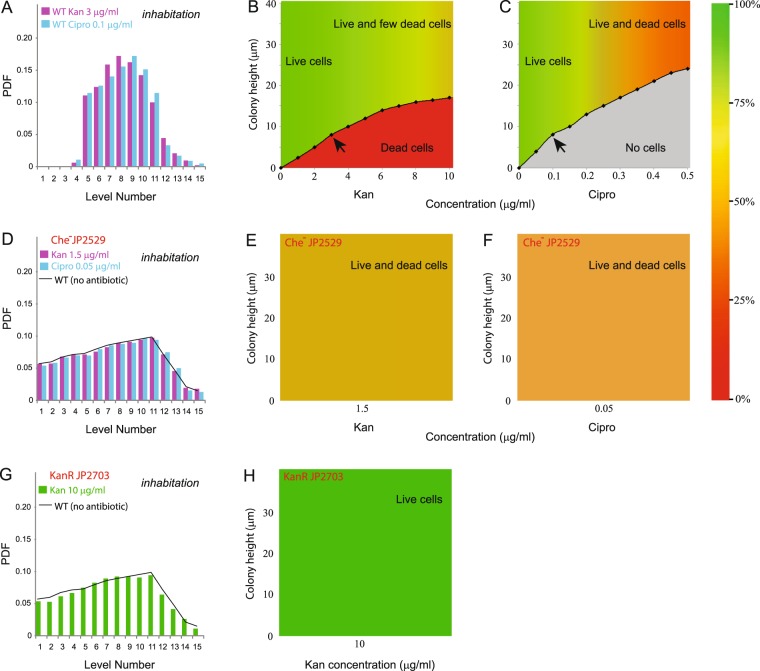
Inhabitation of WT and Che- bacteria within the swarm in the presence of antibiotics. Cells were inoculated directly on the swarm agar containing antibiotics and tracked by DI as in Fig. 2. (**A**) In the presence of indicated concentrations of Kan and Cipro, the WT strain showed absence of bacteria in levels 1 to 4 (~8–11 μm). PDF values represent >100,000 data points for each antibiotic. (**B,C**) The experiment in (**A**) was repeated with a range of antibiotic concentrations but with a non-fluorescent WT strain, and cells were observed using Live-Dead staining, where live cells show green fluorescence and dead cells show red fluorescence (see Methods). The color chart on the right indicates % of live cells. The MIC (μg/ml) for Kan and Cipro for *S. marcescens* in liquid vs. swarm was reported to be <5 vs. >20 and <0.025 vs. >0.5, respectively^18^. (**D**) Cell occupancy of the fluorescent Che^-^ strain JP2529 was monitored in a swarm with the indicated antibiotics present. The habitation pattern resembled that of WT on antibiotics-free agar (black trace). (**E,F**) Live-dead staining of non-fluorescent Che^-^ JP2529 cells under antibiotic conditions as in D. (**G**) A fluorescent Kan^R^ strain (JP2703_GFP_) shows an inhabitation pattern indistinguishable from that of WT on antibiotics-free agar (black trace). (**H**) Live-dead staining of non-fluorescent Kan^R^ JP2703 cells under Kan concentrations identical to that shown in (**D**).

Bacteria in a swarm can survive concentrations of antibiotics lethal to the same bacteria in bulk liquid^18^. Preliminary experiments showed that this tolerance was lost if the bacteria could not perform chemotaxis, implicating a role for the chemosensory system in the tolerance response (Fig. S3). In order to compare the 3D structure of WT vs Che^−^ swarms, we titrated antibiotic concentrations down to levels where the WT would initiate swarming when inoculated from the center of the plate, as was done for the experiments shown in Figs 2 and 3. Swarming was initiated with a mixture of unlabeled and fluorescently labeled cells on plates that contained the indicated low levels of Kan and Cipro, and cell occupancy measured by the DI methodology in a similar 3D region of the swarm as before. Colony height in this area remained ~40 μm. Analysis of cell speeds and cell trajectories in a WT swarm, showed these properties to be almost independent of the colony height (Fig. S4). In WT, the most significant change in swarm structure was observed at the bottom boundary near the agar at levels 1–4, close to the source of the antibiotic (Fig. 4A). This zone was depleted of fluorescent cells, as inferred by the absence of diffraction rings with expected diameters corresponding to the *z* positions for these levels (see Movie S6). This suggested that there were either no live cells in this zone, or that there were dead cells that did not fluoresce.

To detect the presence of dead cells, we added a Live-Dead stain to the medium, which stains live cells green and dead cells red (see Methods). For these experiments we used a strain without the GFP plasmid, and followed the distribution of red and green cells over a larger range of antibiotic concentrations. We indeed observed a zone of dead bacteria at the bottom with Kan, which matched the zone that was devoid of fluorescent (live) cells seen in Fig. 4A; the greater the Kan concentration, the larger the dead-cell zone (Fig. 4B). The results were slightly different for Cipro, where the depletion zone at the bottom had no dead cells; here, dead cells (which were more elongated) were instead found mixed with the live cells throughout the height of the swarm (Fig. 4C). We conclude that bacteria in a swarm migrate away or avoid the bottom antibiotic boundary. The avoidance zone is seen with both antibiotics, but the presence or absence of dead cells in this zone is antibiotic-specific and may be related to a secondary effect of the antibiotic on cell morphology.

### The antibiotic avoidance response at the bottom boundary is lost in a chemotaxis-defective mutant

The sensitivity of the Che^−^ mutant to antibiotics (Fig. S3) suggested that WT bacteria may employ the chemosensory system for avoiding the bottom agar boundary, where antibiotics are present. To test this possibility, we had to work at the lower end of the antibiotic range shown for WT in Fig. 4B,C in order to keep other experimental conditions the same. We observed that even at this lower range, the avoidance zone at the bottom boundary seen with the WT (Fig. 4A) was lost in the Che^-^ mutant for both antibiotics (Fig. 4D). Live-Dead staining showed that both kinds of cells were mixed through all the levels (Fig. 4E,F). We conclude that the chemotaxis signaling system plays a role in the avoidance response of the WT swarm, in the absence of which, cells become more sensitive to antibiotics (Fig. S3).

### Avoidance behavior is not a response to the antibiotic per se

Dependence of the avoidance response (Fig. 4A) on a functional chemotaxis pathway (Fig. 4D) might implicate the antibiotic in directly stimulating a chemosensory response by binding to the chemoreceptors (see Fig. S1). However, there are no reports to date of such a response in bacteria. An alternate possibility is that the avoidance response is induced by chemicals released when cells are killed by the antibiotic (Fig. 4B). To distinguish between these alternatives, we constructed a strain (JP2703) that would not be killed by Kan due to the introduction of a plasmid (pBAD18-Kan) specifying Kan resistance^34^. The resistance arises by intracellular modification (and inactivation) of the antibiotic by an enzyme encoded in the plasmid. Since ligands are detected in the periplasm, kanamycin might be expected to induce a signaling response during its uptake into the periplasm, prior to entry and inactivation in the cytoplasm^35^. We observed that at 10 μg/ml Kan, where WT cells avoid the bottom boundary (Fig. 4A), JP2703 did not do so, and showed a cell distribution pattern similar to WT in the absence of the antibiotic (Fig. 4G). No cell death was observed for JP2703 under these conditions, as expected (Fig. 4H). These results suggest that cell migration away from the bottom boundary is unlikely to be due to a direct response to the antibiotic, and favor the possibility that cell death contributes to the response indirectly.

## Discussion

This work provides the first view of the 3D architecture of a bacterial swarm and the individual cell behavior within. We find that in a *S. marcescens* swarm, the hallmark swirling motion of the swarm takes place mostly in an intermediate zone, away from the solid-liquid boundary at the bottom and the liquid-air boundary at the top. There is a gradient of cell density from the bottom to the top, with maximal occupancy around two-thirds of the colony height, the cell population abruptly dropping off approaching the top. This architecture is not dictated by chemotaxis, as it was also seen in a chemotaxis-defective mutant. However, this density profile is clearly dependent on the presence of the surfactant secreted by *S. marcescens*, because both occupancy and speeds became uniform in the surfactant-defective mutant. The swarm architecture reorganizes at the bottom boundary when antibiotics are introduced.

Low concentrations of surfactants can form a monolayer at liquid-air interfaces or aggregate into larger micelles at higher concentrations^36,37^. The driving force for micelle formation depends on the structure of the amphiphile, and the interplay between hydrophobic and hydrophilic interactions of the molecules, which depend on conditions such as temperature, presence of salt, pH etc. Our observations suggest a previously unknown interaction between the active bacteria and a surfactant-laden liquid-air interface. This interaction prevents cells from accumulating near the surface (Fig. 2). The depletion zone at the upper boundary disappeared in the Srw^−^ mutant and reappeared upon addition of surfactin, a biosurfactant from *B. subtilis*, which like serrawettin, is also a lipopeptide^5^ (Fig. 3). Placing a glass cover at the top also displaced the surfactant and consequently eliminated the depleted zone (Fig. 3). Other evidence supporting this new interaction at the upper levels is our observation that cells were occasionally immobilized in the upper layers with almost no movement, indicating the presence of material other than water/fluid (see Movie S2). These cells were not immotile, because they were capable of being released, migrating down back to the colony where they resumed normal movement. The exact physical nature of this interaction is beyond the scope of the current paper. Nonetheless, we hypothesize that the depleted layers are either due to formation of a thick micelle layer or repulsion from a new form of effective image dipoles across the surfactant-laden boundary.

At the liquid-air upper boundary of WT swarms, the speed of cells dropped to zero (Fig. 2). In the absence of surfactant, or when the surfactant layer was displaced with glass, this behavior was no longer seen (Fig. 3), suggesting that the boundary condition to the velocity is a Neuman (no-flux) condition i.e., the component of the velocity gradient normal to the interface vanished. This implies that for surfactant-secreting swarming bacteria (and possibly for some other systems of self-propelled particles such as active colloids), the appropriate boundary condition is the exact opposite of that expected by standard fluid dynamics. For inert, Newtonian fluids, one expects a vanishing boundary condition for a glass interface and a no-flux boundary with air. Because the characteristic distance for the fluid boundary layer is significantly larger than the cell thickness, it is not surprising that the boundary condition for WT swarming cells is different than fluid flow. Another curious observation was that there was no accumulation of bacteria at the upper boundary when glass was introduced (Fig. 3C), a contrast to 3D swimming bacteria that tend to accumulate near glass surfaces, where they move in low-curvature circles^28,29,38–43^. This accumulation has been attributed to an effective hydrodynamic interaction with an image hydrodynamic dipole^38^. In a 3D swarm, on the other hand, the bacteria respond very differently at both the boundaries. It is possible that the high density of the swarm lowers the viscosity of the fluid, effectively screening hydrodynamic repulsion.

The most surprising observation in this study is that in a 3D swarm, the bacteria migrate away from the bottom boundary and create an avoidance zone when faced with antibiotics (Fig. 4A–C). Such a response has not been reported in 3D swimmers, while in 2D swarms, antibiotics were reported to create a motility-defective sub-population that self-segregated into clusters^8^. We had speculated earlier that the improved ability of the swarm to survive antibiotics might be related to the presence of dead cells in the population; these cells might offer a physical barrier between the antibiotic source and the live cells, or release a warning signal to the live cells^18^. Dead cells were indeed observed in the avoidance zone, but only with one of the two antibiotics tested, ruling out dead cells as a general physical barrier (compare Fig. 4B,C). Instead, we observed a dependence of the avoidance zone on the presence of a functional chemotaxis signaling pathway (Fig. 4D), which is known to respond to several repellents such as heavy metal ions, some hydrophobic amino acids, weak acids, and membrane disruptive compounds such as indole. In the absence of a functional chemotaxis pathway, the swarm also became more sensitive to the antibiotics (Fig. S3), suggesting that this pathway contributed to its high antibiotic tolerance. To date however, there is no report that bacteria perceive antibiotics as repellents. Absence of an avoidance response to Kan in a Kan^R^ population where there is no cell killing (Fig. 4G,H), shows that the antibiotic is indeed not sensed directly as a ligand by the chemoreceptors, and favor the hypothesis that cell killing releases a ‘signal’ that triggers the repellent response.

In summary, this study takes a first look inside a 3D swarm, and finds a novel architecture to the colony, where a biosurfactant layer occupies a significant region of the space on top of the colony, keeping bacteria away from the swarm-air upper boundary. Maximal cell occupancy is at an intermediate region between the upper and lower swarm boundaries. This arrangement may offer maximal protection from environmental assaults at both boundaries. The bacteria can create an avoidance zone when antibiotics are added at the lower boundary, a phenomenon that depends on a functional chemosensory pathway, and may contribute to the high tolerance of the swarm to antibiotics.

## Methods

### Bacterial strain and growth protocol

All strains and plasmids used in this work are listed in Table S1. Experiments were predominantly performed with wild-type (WT) *Serratia marcescens* 274, which is a Gram-negative rod-shaped (0.8 × 4 μm on swarm agar) flagellated species, used as a model system in previous quantitative swarming (and collectively swimming) experiments^10,11,44,45^. The cells were grown on agar plates consisting of 25 g/l LB (Luria broth, Sigma, St. Louis, MO), and 0.5% agar (Difco Franklin Lakes, NJ) for swarm conditions, and 0.3% agar for swim/chemotaxis assays, both at 30 °C with 95% RH (Relative Humidity). Each plate contained 20 ml of growth medium. Plates were allowed to dry for 24 h after pouring, before inoculating the center with 5 μl of cell culture grown overnight in liquid medium. The Che^−^ mutant JP2529 and the Srw^−^ mutant RH1041 required a 50 μl initial inoculum, as did strains whose 3D structure was monitored in the presence of antibiotics added to the growth medium; the 95% RH in the incubator also facilitated swarming in these strains. For restoration of surfactant to the Srw^−^mutant of *S. marcescens*, 1 μg of *B. subtilis*-derived surfactin (purchased from Sigma) was added to the 5 μl inoculation drop. Plasmids were maintained with either 100 μg/ml ampicillin or 50 μg/ml kanamycin added to the medium, unless indicated otherwise.

The Che^−^ strain JP2529 harboring a targeted disruption of the *cheY* gene by a Cm (Chloramphenicol) cassette was constructed as follows. The Cm cassette was amplified by PCR from pKD3^46^ along with flanking regions of ~400 bp homologous to the upstream and downstream regions of the *S. marcescens cheY* gene using appropriate oligonucleotides. ~1 μg of this *cheY*::Cm disruption cassette was electroporated into WT *S. marcescens* carrying the plasmid pKOBEGA for lambda red recombinase expression^47^. Mutants were selected on chloramphenicol (1000 μg/ml), confirmed by PCR and DNA sequencing, as well by observation of a smooth swimming (run-only) phenotype under a microscope (Fig. S1A). To induce motor reversals (tumbling behavior during swimming) in JP2529, plasmid pXYZ202 was introduced. This plasmid carries a constitutively active version of *E. coli* CheY (CheY**) expressed from the *trp* promoter, and was gift from Birgit Scharf (Virginia Tech)^48^. Leaky basal expression of CheY** is sufficient to cause tumbling (Fig. S1A). Non-chemotactic mutants of some bacteria, including *S. marcescens*, have been reported to require either more surface hydration or some degree of motor reversals to support swarming^48,49^. Under our experimental conditions (95% RH and 0.5% swarm agar), JP2531 strain swarmed like WT (consistent with its motor reversal frequency being similar to WT; Fig. S1A), while JP2529 (defective in motor reversals) needed a larger inoculation volume (50 μl instead of 5 μl) to do so (see above).

To follow the motion of single cells within the dense swarm, a plasmid encoding a green fluorescent protein (pTRC99a::GFP) was introduced into all the strains used for tracking. GFP expression was induced with 50 μM IPTG. Unlabeled cells were mixed with GFP-expressing cells at a ratio of ~1000:1 and co-inoculated on swarm plates (without addition of ampicillin). GFP does not affect swarming behavior, colony expansion speed or growth rate^10^. Cells were tracked in 3D after 2 h of growth, when the swarm colony diameter was ~4 cm. The agar plate was placed directly on the microscope stage and viewed from the top.

The fluorescent KanR strain of *S. marcescens* (JP2703_GFP_) was created by first introducing pBAD18-Kan into the WT strain (JP2703), followed by the GFP-encoding plasmid.

### Microscopy

Optical microscopy (Zeiss Axio Imager Z2), equipped with a sensitive high resolution video camera (NEO, Andor), was used to capture the motion of the fluorescently labeled cells (33.33 frames/s and 1800 × 1800 pixels). Swarm plates were placed on the microscope stage and were viewed from the top. Cell trajectories were obtained and analyzed using Matlab. No photobleaching was observed during the acquisition times of one minute for each experiment (2,000 frames). Each field of view typically had a few tens of labeled cells at 63X magnification. The total data summarizes results from tens of experiments with hundreds of cells followed in each experiment, with a final yield of >100,000 data points. Because standard fluorescent light strongly affects cell motility (it usually completely stops their motion in less than 1 s), we used a modified version for the filters and dichroic mirror. The GFP-labeled cells were observed by standard yellow fluorescent protein (YFP) Zeiss illumination setup instead of the standard green GFP one (Filter set 46 YFP shift free: Excitation 500/25; Beam Splitter 515; Emission 535/30); the cell intensity was slightly weaker compared to GFP.

### Three-dimensional tracking

The system was operated in an off-focus-fluorescence mode where cells located at the lower levels of the colony i.e., on the agar, are in full focus, while the cells in upper layers form diffraction rings at an increasing diameter depending on how high (close) they are to the lens;^31^ the higher the cell, the larger the diffraction ring. Calibration of the diffraction ring diameter as a function of cell position on the *z*-axis was achieved as described previously^31^, by embedding immobile spherical particles (carboxylate-modified microspheres, 1 μm in diameter; Molecular Probes) at different heights in agar, then changing the sample’s height precisely with a 0.01 μm resolution (a feature of the Axio Imager Z2) and measuring the diameter of the diffraction ring. We ascertained that rod-shaped cells in a crowded environment had little effect on the shape of the diffraction ring by repeating the calibration with GFP-labelled immotile cells (RH1037) mixed at ratio of 1:1000 with immotile non-labeled cells and grown on agar.

A semi-automated Matlab program was created to obtain the bacterial trajectories and calculate speeds. The user recognizes a ring, then the center of the ring is chosen manually and its coordinates are saved for every frame along the movie yielding the *x-y* position of the cell as a function of time. The changing diameter of the ring is measured by following its perimeter at four locations (up, down, left and right, then averaging); the diameter is converted to the *z* position according to the calibration curve. The process is repeated for all rings in the movie. Each ring (cell) was followed typically for few seconds until it left the field of view. The 3D trajectories were smoothed using Matlab’s malowess function which locally fits a polynomial (2nd order) to a moving window (7 frames); then the velocities, curvature and torsion were calculated.

For a parallel estimation of colony height, small MgO smoke particles (~0.5 μm in diameter) were gently placed on top of the colony^11^. The height was measured by directly focusing on the particles (phase contrast), then moving the stage towards the colony edge and focusing on the agar. The microscope yields a precise (±0.01 μm) read of the difference in height on its screen. The value was same as the one obtained for the colony height using the off-focus fluorescence technique using the diffraction rings with the carboxylate-modified 1 μm beads.

### Live-dead staining

To test for the presence and distribution of dead bacteria, a mix of 30 μM propidium iodide (stains dead cells red) and 24 μM STYO-9 dye (stains live cells green), (obtained from Molecular Probes; Kit L13152) dissolved in double-distilled water. A 10 μl of the mix was added to the agar at the center of the plate 2 h before cell inoculation. With no added dye, 1% of the population self-fluoresce as red (no self-fluorescence in green); with added dye, 3% of the cells were red (dead) even without antibiotics. Dead cells were located in the colony along the *z*-axis by traveling with the microscope stage, looking for red-labelled cells to be in focus. The dead/live staining experiment was performed in the absence of GFP-labeled cells.

### Border crossing assay

Border-crossing experiments were carried out as described^18^, utilizing dual-compartment petri dishes with 30 mL of LB swarm media added to each chamber. Antibiotic was only added to the right chamber. Before the media hardened, a sterile pipette tip was used to drag the meniscus over the plastic border, connecting the two sides with a ~1 mm tall agar bridge. Plates were inoculated in the left chamber with 5 μl of cell culture as described above for 3D tracking experiments, and photographed using a Canon Rebel XSI digital camera.

### Bead assay for flagellar motor behavior

Briefly, flagellar filaments were sheared off, and cells fixed to a glass slide, before 0.75 µM polystyrene beads were attached to the remaining filament stubs as described^50^. High-speed video of individual beads was captured with a CCD camera (ICL-B0620M-KC0; Imperx, Boca Raton, FL) at 1,250 frames/s, and bead rotation angular speeds and directional changes were used as an indicator of motor function.

### Curvature and torsion of trajectories

Turns and twists of 3D trajectories can be described in terms of the curvature and the torsion. Intuitively, the radius of curvature is the radius of the circle that locally approximates the curve. Mathematically, let $x(s)$ denote a differentiable 3D curve parametrized by arc length *s*. The curve specifies a local orthogonal coordinate system given by the tangent vector, ${t}^{\text{'}}={x}^{\text{'}}/|{x}^{\text{'}}|$, the principal normal vector $n={t}^{\text{'}}/|{t}^{\text{'}}|$ and the binormal vector $b=t\times n$. Then, the curvature of the curve at point *s* is the magnitude of the rate of change of the tangent vector $\kappa =|t\text{'}(s)|$. The radius of curvature *R* is $R=1/\kappa$. The torsion describes twists in the trajectory and is given by $\tau =-\,n\cdot b\text{'}$.

Figure 2G shows the probability distribution of finding significant curvatures (>1 µm^−1^) and torsions (>1 rad/µm) in the trajectories of the cells (by significant we mean non-negligible or non-noisy). The graph is normalized (sum = 1). Hence, it shows the relative probability for a large curvature/torsion in each level. These two quantities define sharp turns and changes in the plane of motion. Choosing different values for the noise threshold (e.g., 0.3, 0.5 or 2.0 which defines how significant they are) did not change the trend of the graphs (unless the values were too large (>2.5) yielding fewer points thus poor statistics). Movie S5 shows the curvature and torsion of a single cell (the one plotted in Fig. 1F–H) as a function of time with the significant events (those other than what is defined as smooth trajectories) marked with dots. In Fig. 2G, for normal conditions (WT), we see that cell trajectory is relatively smooth in the intermediate levels of the colony where intensive collective motion occurs (see Movie S4), but that close to the boundary the cells change their direction of motion abruptly. Similarly to our previous findings, chemotaxis mutants yielded same results (Fig. 2H). In Fig. 2I we plot (on a log-log scale) the distribution of steps between significant curvatures (>1 µm^−1^) and torsions (>1 rad/µm) events in the trajectories of the cells (for normal conditions). The results suggest that the distribution has a power-law tail, which is different from the exponential distribution obtained for cells swimming in bulk liquid. This suggests that, similarly to 2D swarms, cells in 3D swarms follow a motion pattern that is consistent with a Lévy-walk^18^. However, our current data are insufficient to establish the precise geometry of the trajectories.

## Electronic supplementary material

Supplementary Information

Movie S1

Movie S2

Movie S3

Movie S4

Movie S5

Movie S6
