# Supplementary material for: The 3D architecture of a bacterial swarm has implications for antibiotic tolerance
Source: Sci Rep. 2018 Oct 25;8:15823. doi: 10.1038/s41598-018-34192-2 (PMC6202419; doi:10.1038/s41598-018-34192-2)
Supplement: Supplementary file 1 — Supplementary Information [file 41598_2018_34192_MOESM1_ESM.pdf]

Supplementary Material

The 3D architecture of a bacterial swarm has implications for antibiotic tolerance

Jonathan D. Partridge[1], Gil Ariel[2], Orly Schvartz[3], Rasika M. Harshey[1,*] and Avraham Be'er[3,4*]

[1]Department of Molecular Biosciences, University of Texas at Austin, Austin, Texas 78712, USA; [2]Department of Mathematics, Bar-Ilan University, Ramat Gan 52000, Israel; [3]Zuckerberg Institute for Water Research, The Jacob Blaustein Institutes for Desert Research, Ben-Gurion University of the Negev, Sede Boqer Campus 84990, Midreshet Ben-Gurion, Israel; [4]Department of Physics, Ben-Gurion University of the Negev 84105, Beer Sheva, Israel;

* Corresponding Authors:

Rasika M. Harshey Tel +1-512-471-6881; Fax +1-512-471-1218; Email rasika@austin.utexas.edu

Avraham Be'er Tel +972-544-337478; Fax +972-8-6563503; Email beera@bgu.ac.il

Supplementary figure, movie and table legends

Figure S1: **The chemotaxis signaling network, bead assay for flagellar motor behavior, and plate assay for chemotaxis. (A)** A schematic of the best-studied *E. coli* chemotaxis signal transduction pathway, expected to be closely related to that of *S. marcescens*. Extracellular ligands (circles) are detected in the periplasm by transmembrane chemoreceptors (MCPs; red) connected to a cytoplasmic kinase (CheA; yellow) via a linker (CheW; blue). A basal rate of autophosphorylation generates CheA~P, which transfers its phosphate to two response regulators (CheB and CheY). CheY~P binds to the flagellar motor, reversing its default counter-clockwise (CCW) rotation to clockwise (CW). CCW states promote runs, while CW states promote tumbles. The phosphatase CheZ dephosphorylates CheY~P, terminating the tumble response. In the absence of chemoeffector gradients, the cells swim in a random walk of runs and tumbles. Chemoattractant binding represses CheA kinase activity, decreasing tumbling probability and extending runs, allowing cells to move up an attractant gradient. CheB~P governs the adaptation response that returns the MCPs to their pre-signaling state by removing methyl groups added to the MCPs by the methyltransferase CheR, so that the MCPs remain responsive to changes in chemoeffector concentration. MCP methylation controls their kinase on-off states. Absence of CheY (see below) is expected to disable signal transduction through this pathway, resulting in a loss of response to chemical ligands detected by the MCPs, and hence loss of chemotaxis. **(B)** Bead assays to monitor flagellar rotation were performed by fixing cells to a glass slide and attaching polystyrene beads to flagellar filament stubs (see Methods). Motor reversals per minute (left) and average angular speeds in hertz (Hz; rotations per second) of flagellar motors of WT *S. marcescens*, *cheY* deletion mutant (JP2529), and JP2529 expressing CheY** from plasmid pXYZ202 (JP2531). The Δ*cheY* mutant by itself does not show motor reversals as expected (left), but is unaffected in motor speed (right). Complementation of the Δ*cheY* strain with CheY** (a constitutively active variant of CheY, i.e. active in the absence of phosphorylation, hence unregulated by the pathway) results in a substantial restoration of motor reversals. **(C)** Chemotaxis assays on 0.3% soft agar plates for strains shown in B. The ability to migrate out from the point of inoculation at the center is a measure of chemotaxis proficiency. The *cheY* mutant is locked in a run-only mode as determined by the bead assay (B) as well as by observation of bacterial motion. Basal expression of CheY** from a *trp* promoter (see Methods) restores motor reversal (B), but does not restore chemotaxis as expected. Plates were incubated at

30°C for 8 h. Images are representative of three biological replicates each carried out in triplicate.

Figure S2: **3D inhabitation of bacterial species that do not secrete surfactants**. Cells were tracked as described in Fig. 1. Occupancy of **(A)** *E. coli*, and **(B)** *Salmonella* as described in Fig. 2. The occupancy patterns in both A and B resemble that of the Srw$^-$ *S. marcescens* strain RH1041 (green traces). PDF values represent >15,000 cells in both A and B.

Figure S3. Border crossing assay showing antibiotic susceptibility/tolerance of WT and Che$^-$ strains of *S. marcescens*. Cells were inoculated in the left chamber without antibiotic and allowed to either swim (0.3% agar) or swarm (0.5% agar) into the right chamber containing indicated concentrations of Kanamycin **(A)** or Ciprofloxacin **(B)** as described in Methods. WT swimmer cells (top panels in A, B) are sensitive to antibiotic concentrations that are tolerated by WT swarmers (middle panels in A, B), as judged by their inability to cross the border at the lowest antibiotic concentration tested. The swarmer cells of the Che$^-$ strain JP2531 (bottom panels A, B) are more sensitive to both antibiotics compared to WT (middle panels). JP2231 was used for this experiment because motor reversals facilitate swarming in non-chemotactic cells. Plates were incubated at 30°C, swarm plates for 16 h, swim plates for 8 h. Images are representative of three biological replicates each carried out in triplicate.

Figure S4. Speed, torsion and curvatures of trajectories in the presence of antibiotics. (A, B) The average speed in the *x*-*y* plane and along the *z*-direction at different heights, respectively, for cells tracked in Fig. 4A in the presence of 3 μg/ml Kan (purple) and 0.1 μg/ml Cipro (blue). **(C)** PDF of curvatures and torsion events for the cells in A, B (>100,000 data points for each antibiotic). The results are independent of the height.

Movie S1: Phase contrast view of the edge of a WT *S. marcescens* swarming colony in the quasi-2D region. In this region, cells are either one or two layers thick. The movie is in real-time. Screen dimension, 100×100 μm.

Movie S2: Fluorescence imaging of a multilayered region of a WT *S. marcescens* swarm. Fluorescently labeled bacteria were mixed with unlabeled bacteria at a ratio of 1:1000. The diameter of fluorescent diffraction rings reports on the *z* position of the cells, larger rings indicative of cell location at higher levels in the colony. Cells in focus are near the agar surface.

One cell with a large diameter (around the 3 o'clock position) appears to be immobilized at the top of the colony. Other descriptions as in Movie S1 legend.

Movie S3: 3D trajectories of WT cells. The trajectories were collected from movies such as that shown in Movie S2, analyzed by a Matlab program as described under Methods.

Movie S4: A phase contrast recording of the swirling bacteria in the intermediate region (levels 3-5) of the colony. The movie is in real-time. Screen dimension, 100×100 μm.

Movie S5: The trajectory of a single cell in the 3D colony. The curvature and torsion are plotted on the right hand side with red and pink dots indicating sharp turns and twists, respectively. See Methods for details.

Movie S6: Fluorescence imaging of a multilayered region of a WT *S. marcescens* swarm in the presence of Kanamycin. Cells were recorded as described in Movie S2. Absence of cells at the bottom levels 1-4 is inferred from the absence of diffraction rings with the diameter calibrated for these levels. For example, the diffraction rings of the moving cells do not shrink into a sphere (compare with Movie S2), indicating that the cells do not go down to the bottom levels on the agar. This movie was selected to show a rare fluorescent cell at the bottom that apparently survived the antibiotic but was barely moving. This cell corroborates our plane of reference for the bottom level.

Table S1: A summary of bacterial strains and plasmids used in this study.

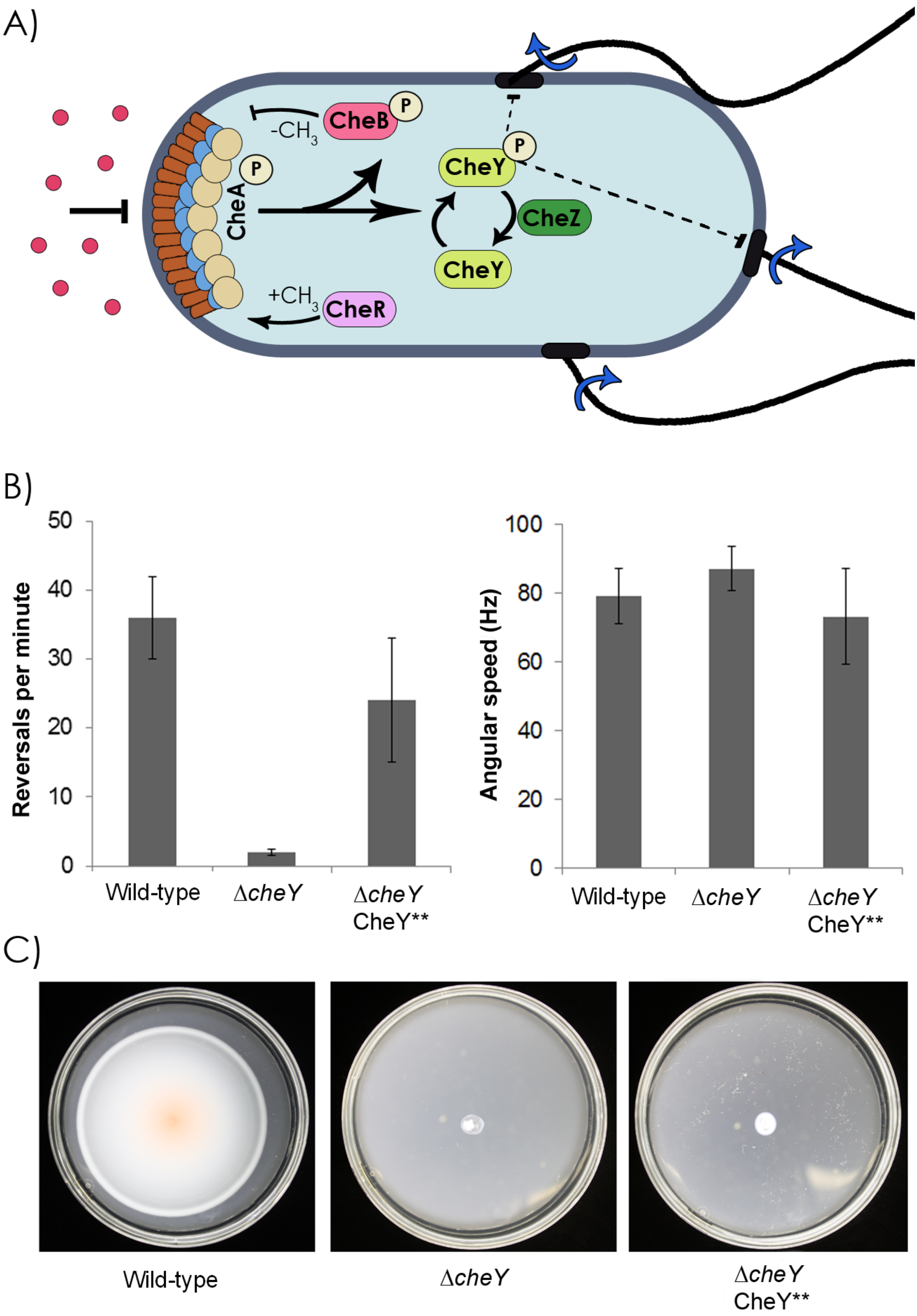
A)
-CH3
CheB
P
CheA
P
CheY
P
CheZ
CheY
+CH3
CheR
B)
Reversals per minute
0
10
20
30
40
50
Wild-type
ΔcheY
ΔcheY CheY**
Angular speed (Hz)
0
20
40
60
80
100
Wild-type
ΔcheY
ΔcheY CheY**
C)
Wild-type
ΔcheY
ΔcheY CheY**

Figure S1

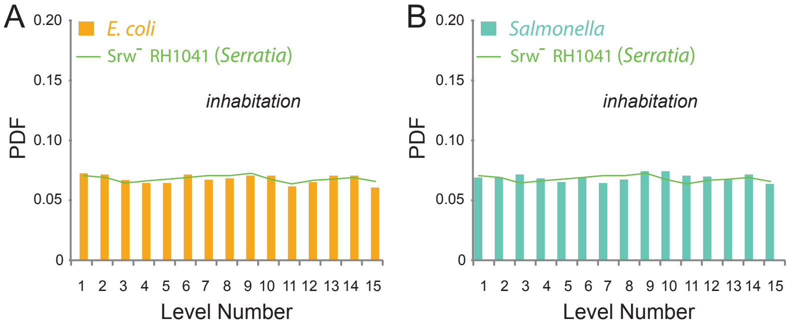
A
E. coli
Srw⁻ RH1041 (Serratia)
inhabitation
PDF
0.20
0.15
0.10
0.05
0
1 2 3 4 5 6 7 8 9 10 11 12 13 14 15
Level Number
B
Salmonella
Srw⁻ RH1041 (Serratia)
inhabitation
PDF
Level Number

Figure S2

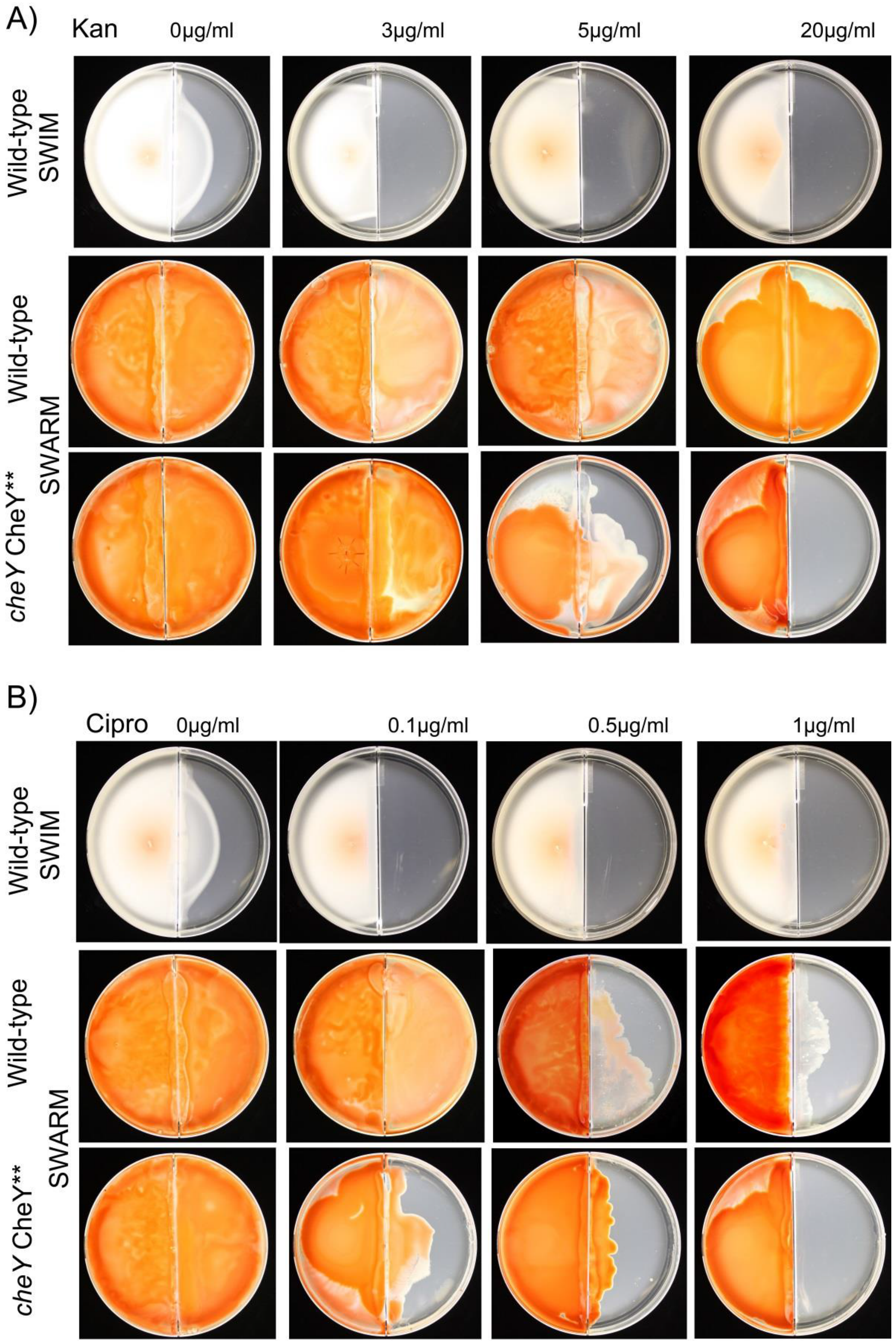
A)
Kan
0µg/ml
3µg/ml
5µg/ml
20µg/ml
Wild-type SWIM
Wild-type
SWARM
cheY CheY**
B)
Cipro
0µg/ml
0.1µg/ml
0.5µg/ml
1µg/ml
Wild-type SWIM
Wild-type
SWARM
cheY CheY**

Figure S3

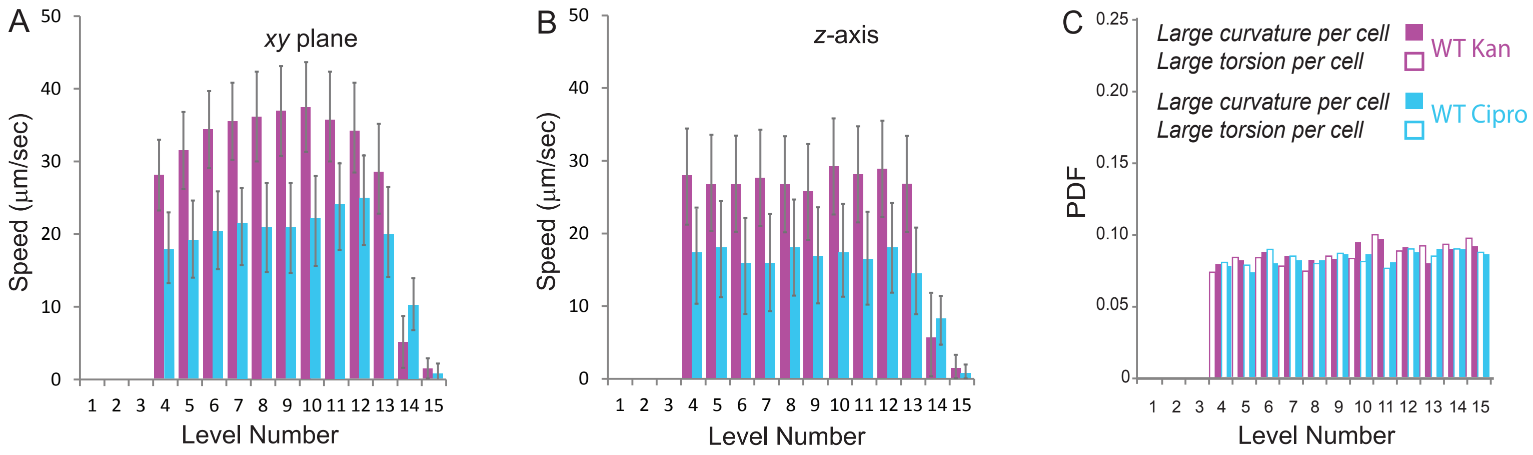
A
xy plane
Speed (µm/sec)
50
40
30
20
10
0
1 2 3 4 5 6 7 8 9 10 11 12 13 14 15
Level Number
B
z-axis
Speed (µm/sec)
50
40
30
20
10
0
1 2 3 4 5 6 7 8 9 10 11 12 13 14 15
Level Number
C
PDF
0.25
0.20
0.15
0.10
0.05
0
Large curvature per cell
Large torsion per cell
WT Kan
Large curvature per cell
Large torsion per cell
WT Cipro
1 2 3 4 5 6 7 8 9 10 11 12 13 14 15
Level Number

Figure S4

Strain	Genotype	Reference
Serratia marcescens 274	*S. marcescens* strain 274 (WT)	1
JP1020	WT *S. marcescens* pTRC99a::GFP	2
JP2529	*S. marcescens cheY*::Cm	This work
$JP2529_{GFP}$	JP2529 pTRC99a::GFP	This work
JP2531	JP2529 pXYZ202	This work
$JP2531_{GFP}$	JP2531 pTRC99a::GFP	This work
JP2703	WT *S. marcescens* pBAD18-Kan	This work
$JP2703_{GFP}$	JP2703 pTRC99a::GFP	This work
RH1037	*S. marcescens* Δ*hag*::Kan (immotile)	3
$RH1037_{GFP}$	RH1037 pTRC99a::GFP	This work
RH1041	*S. marcescens* Serrawettin- ::Kan	4 [as SMu4e]
$RH1041_{GFP}$	*S. marcescens* Serrawettin- ::Kan pTRC99a::GFP	This work
MG1655	Wild type *E. coli* F- λ- ilvG- rfb-50 rph-1	Lab stock
$MG1655_{GFP}$	MG1655 pTRC99a::GFP	Lab stock
14028	Wild type *Salmonella* strain	Lab stock
14028_{GFP}	14028 pTRC99a::GFP	Lab stock

Plasmid		
pKD3	Source for Cm^R cassette	5
pKOBEGA	Lambda Red recombinase plasmid, Ap^R	6
pTRC99a	IPTG-inducible expression vector, Ap^R	Lab stock
pTRC99a::GFP	GFP-expressing pTRC99a	This work
pXYZ202	*E. coli cheY*** expressed from P_{trp} on pBR322 vector, Ap^R	7
pBAD18-Kan	Arabinose-inducible expression vector – source of Kan^R	8

References:

1 Alberti, L. and Harshey, R. M. Differentiation of Serratia marcescens 274 into swimmer and swarmer cells. *J. Bacteriol.* **172**, 4322-4328. (1990).

2 Ariel, G., Rabani, A., Benisty, S., Partridge, J. D., Harshey, R. M. & Be'er, A. Swarming bacteria migrate by Lévy Walk. *Nat. Comm*. **6**, 8396 (2015).

3 O'Rear, J., Alberti, L. and Harshey, R. M. Mutations that impair swarming motility in *Serratia marcescens* 274 include but are not limited to those affecting chemotaxis or flagellar function. *J. Bacteriol.* **174**, 6125-6137.

4 Matsuyama, T., Bhasin, A., Harshey, R.M. Mutational analysis of flagellum-independent surface spreading of *Serratia marcescens* 274 on a low-agar medium. *J. Bacteriol.* **177**, 987–991.

5 Datsenko, K. A & Wanner, B. L. One-step inactivation of chromosomal genes in *Escherichia coli* K-12 using PCR products. *Proc. Natl. Acad. Sci. USA*. **97**, 6640–6645 (2000).

6 Chaveroche, M. K., Ghigo, J. M. & d'Enfert, C. A rapid method for efficient gene replacement in the filamentous fungus *Aspergillus nidulans*. *Nucl. Acid. Res*. **28**, 22 e97 (2000).

7 Mariconda, S., Wang, Q. & Harshey, R. M. A mechanical role for the chemotaxis system in swarming motility. *Mol. Microbiol*. **60**, 1590–1602 (2006).

8 Guzman, L.M., Belin, D., Carson, M.J., and Beckwith, J. Tight regulation, modulation, and high-level expression by vectors containing the arabinose PBAD promoter. *J. Bacteriol.* **177**, 4121-4130 (1995).
